# Occurrence of *Neobenedenia girellae* (Monogenea: Capsalidae) in Gilthead Seabream *Sparus aurata* (Actinopterygii: Sparidae) Cultured in Portugal

**DOI:** 10.3390/pathogens10101269

**Published:** 2021-10-01

**Authors:** Perla Tedesco, Monica Caffara, Nuno Miguel Ribeiro Moreira, César Gomes, Andrea Gustinelli, Maria Letizia Fioravanti

**Affiliations:** 1 Department of Veterinary Medical Sciences (DIMEVET), Alma Mater Studiorum University of Bologna, Via Tolara di Sopra 50, Ozzano Emilia, 40064 Bologna, Italy; monica.caffara@unibo.it (M.C.); andrea.gustinelli2@unibo.it (A.G.); marialeti.fioravanti@unibo.it (M.L.F.); 2 MVAQUA, Av. Parque de Campismo, Lt 24, Fr C, 3840–264 Gafanha da Boa Hora, Portugal; nunoribeiro.mvaqua@gmail.com; 3 Aquabaía, Ribeira Brava, 9350 Madeira, Portugal; contabilidade@aquabaia.pt

**Keywords:** Capsalidae, *Neobenedenia girellae*, morphology, genetics, gilthead seabream, *Sparus aurata*, aquaculture

## Abstract

Monogenean capsalids of the genus *Neobenedenia* are widespread parasites of wild and farmed marine fish, and represent a potential threat to mariculture due to their pathogenicity and ability to cause mortality in fish maintained in controlled conditions. The identification of *Neobenedenia* species and, consequently, the definition of their host specificity is often problematic due to their highly conserved morphology; therefore, in order to establish their specific identity, microscopic observation should be complemented with molecular analysis. The present work aims at characterizing *Neobenedenia* specimens infecting the skin of cage reared gilthead seabream *Sparus aurata* from Portugal. Parasite samples obtained from caged fish were processed for morphological analysis, through observation in light and scanning electron microscopy, and for molecular analysis, through amplification and sequencing of 28S rDNA and *cytB*, aimed at identifying them to the species level. Our results showed that the collected parasites belonged to the species *Neobenedenia girellae*; the susceptibility of *S. aurata* towards this pathogenic capsalid monogenean highlighted in the present work represents an important risk in the farming of this valuable fish species.

## 1. Introduction

Capsalids are a cosmopolitan family of monopisthocotylean monogeneans parasitizing the skin, gills, eyes, nasal cavities and fins of farmed and wild marine fish [1]. This family includes pathogenic species capable of causing epizootics in fish farms [2]; among these, members of the genus *Neobenedenia* can be highly pathogenic and are sometimes lethal to farmed fish, while there is no report of pathology associated with these parasites in wild hosts. Due to their low host specificity, wide distribution and ability to cause mortality when present at high intensity, *Neobenedenia* spp. represent a relevant threat to mariculture [3].

*Neobenedenia* spp. have direct life cycles with short generation times [4]: adults continuously lay filamentous eggs that hatch oncomiracidia, the infectious free swimming ciliated larval stages. Therefore, even isolated individuals may represent a potential health threat to fish kept in captivity [5].

During initial infections, the parasite attaches preferentially on the fins and over the cranial skin region, while in long term infections it occurs mainly over ventral and dorso-lateral skin regions [6,7,8]. In the fish host, lesions are produced by the attachment of the parasite and its active feeding on mucus and epithelial cells, and include skin ulcers, particularly over the head region [9] and eye damage [10]. Secondary bacterial infection may also easily occur in the produced lesions [11]. In farmed fish, the parasite is also reported to affect fish growth performance [6,12]. Recent studies conducted on the greater amberjack *Seriola dumerili* showed a significantly lower condition factor and higher feed conversion ratio with increasing number of parasites [6] and a significantly reduced growth of infected individuals compared to uninfected ones [12].

Morphological features important for the correct classification of members of the genus *Neobenedenia* include features of the reproductive system (particularly the lack of a vagina) and of the haptor (path of tendons, sclerite morphology), and shape and form of the eggs [1]. Accurate identification of *Neobenedenia* species based on the mere morphology is challenging [13], but the integration of morphological studies with molecular methods based on the analysis of nuclear and mitochondrial markers is helping to resolve the taxonomy of this genus, allowing identification of the species *N. girellae* (Hargis, 1955) Yamaguti, 1963 as the primary parasitic monogenean in worldwide aquaculture and to separate it from the morphologically indistinguishable *N. melleni* (MacCallum, 1927) Yamaguti, 1963 [14]. In fact, both species exhibit low host specificity, being reported from several farmed fish species, such as the greater amberjack *S. dumerili*, the yellowtail amberjack *Seriola quinqueradiata*, the tiger puffer *Takifugu rubripes*, the Japanese flounder *Paralichthys olivaceus*, the cobia *Rachycentron canadum* and the spotted halibut *Verasper variegatus* [3,10,11,12,15,16]. In the farming of some of these species, particularly the greater amberjack, *N. girellae* causes high mortality and represent a main production bottleneck [8,17].

The gilthead seabream *Sparus aurata* Linnaeus, 1758 is one the main farmed fish species in European aquaculture and is subjected to multiple parasitic infections throughout its production cycle [18,19]. Colorni [20] reported the presence of *N. melleni* in *S. aurata* farmed in concrete tanks in Eilat, Red Sea; particularly, he described the hyperparasitization of *N. melleni* with the dinoflagellate *Amyloodinium ocellatum* Brown, 1931, however a detailed description of the parasite morphology and its infection pattern in the fish was not provided in the report. In a more recent work, specimens of *Neobenedenia* infecting *S. aurata* from the same area were analyzed using different molecular markers and identified as *N. girellae* [14]. No other data is available in the literature on *Neobenedenia* spp. infestations in *S. aurata*.

The present work provides morphological and molecular evidence of the occurrence of *N. girellae* in cage-reared gilthead seabream from a Portuguese farm, confirming the susceptibility of this fish species towards this parasite.

## 2. Results

Capsalid monogeneans referable to the genus *Neobenedenia* were recovered from the skin of 49 parasitized gilthead sea bream from facility A (prevalence of 24.0%) and five parasitized gilthead sea bream from facility B (prevalence of 20.8%) with a mean intensity of 4.96 ± 3.85 (Min 1–Max 33) and 1.40 ± 0.75 (Min 1–Max 2), respectively. In facility A the intensity of infection showed a double value in fish weighing >30 g compared to that of fish ≤30 g (7.5 vs. 3.7). Again, in facility A the intensity was slightly higher in moribund fish than in the randomly collected (5.3 vs. 4.25), while in facility B the parasites were found only in a single cage from moribund fish.

Macroscopical external lesions were either not present in parasitized fish, or difficult to establish given the concomitant presence of infectious disease such as LCDV in fish less than 30 g. The parasites were mostly observed on the flanks of the fish, but also on the operculum and attached to the ocular surface. Morphological characterization was carried out on a total of 15 specimens, following Whittington and Horton [1].

### 2.1. Morphological Description

**Figure 1:**
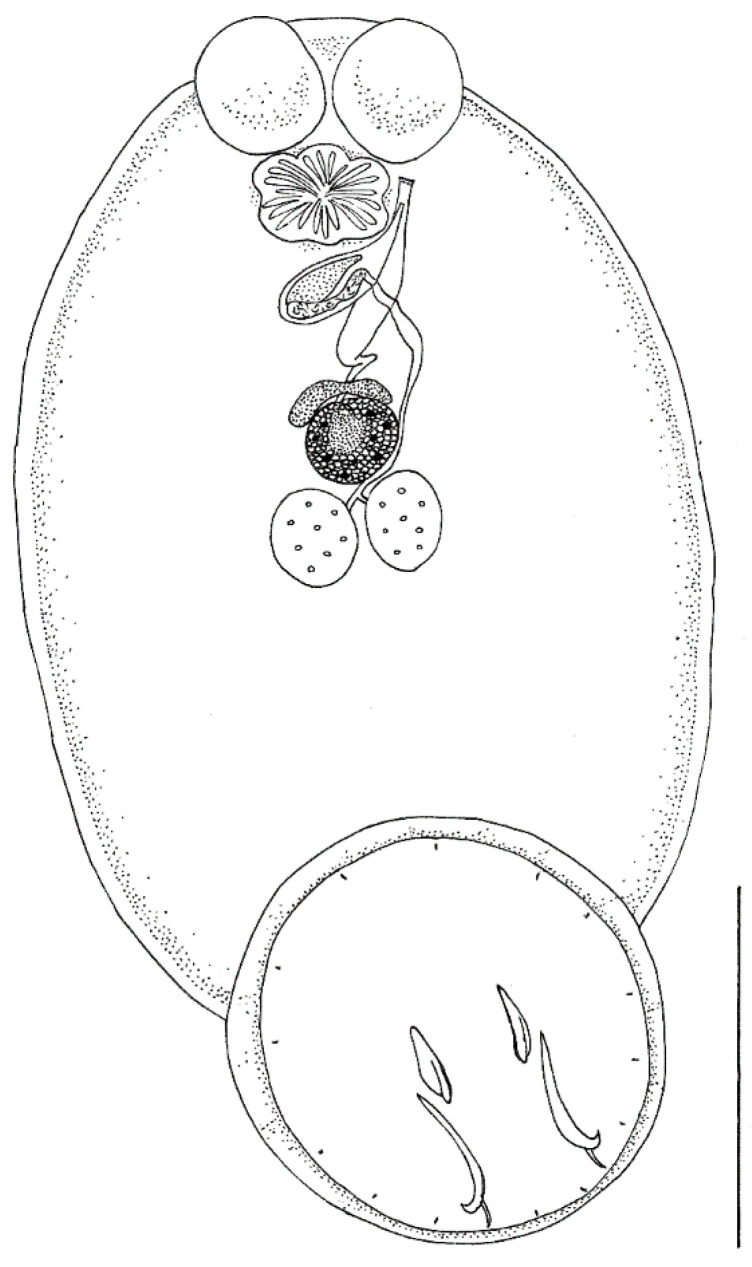
*Neobenedenia girellae* adult from *Sparus aurata*: line drawing. Scale bar = 1000 µm.

**Figure 2:**
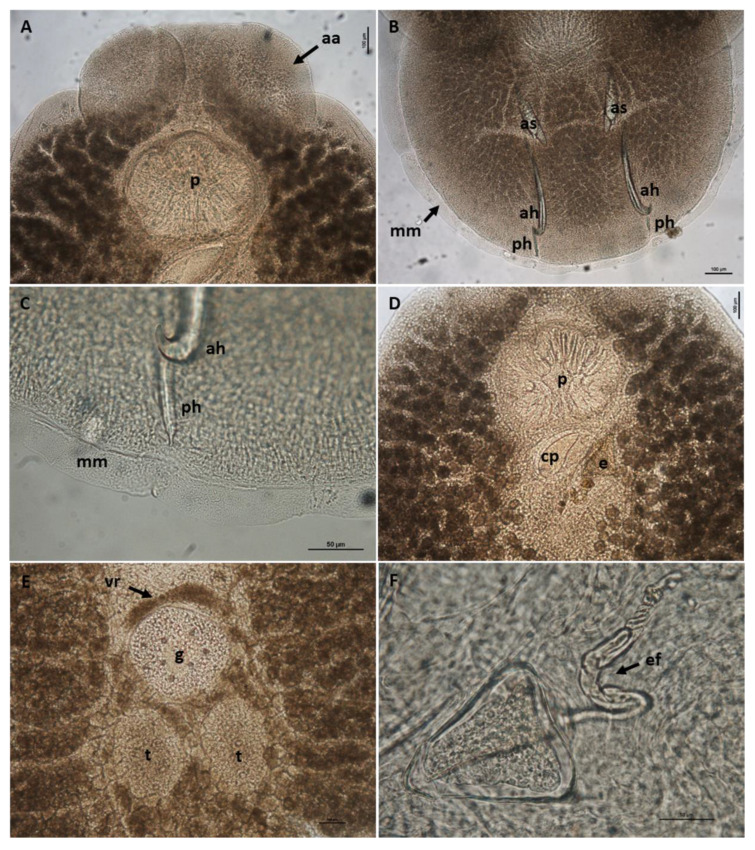
*Neobenedenia girellae* adult from *Sparus aurata*. (**A**) anterior end with disc-like anterior attachment organs (aa) and lobed pharynx (p), scale bar = 100 µm; (**B**) haptor with accessory sclerites (as), anterior hamuli (ah), posterior hamuli (ph) and marginal membrane (mm), scale bar = 100 µm; (**C**) detail of the marginal membrane and the distal part of anterior and posterior hamule, scale bar = 50 µm; (**D**) cirrus complex within cirrus pouch (cp) and egg (e), scale bar = 100 µm; (**E**) central part of body with testes (t), germarium (g) and vitelline reservoir (vr), scale bar = 100 µm; (**F**) detail of the egg with coiled egg filament (ef), scale bar = 50 µm.

**Figure 3:**
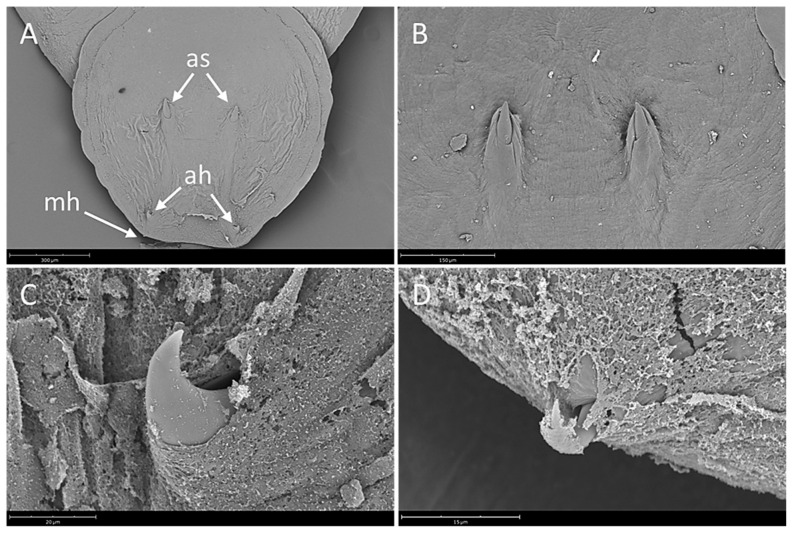
*Neobenedenia girellae*, SEM micrographs. (**A**) haptor showing a pair of accessory sclerites (as), a pair of anterior hamuli (ah) and marginal hooklet (mh), scale bar = 300 µm; (**B**) detail of accessory sclerites, scale bar = 150 µm; (**C**) detail of anterior hamulus, scale bar = 20 µm; (**D**) detail of marginal hooklet, scale bar = 15 µm.

Body elongate oval, total body length including haptor 4207.3 ± 1583.4 (3339.1–5464.0), maximum width 2021.8 ± 364.4 (1283.4–2500.4) (Figure 1). A pair of anterior attachment organs almost circular, 458.2 ± 199.3 (123.6–616.7) long by 391.1 ± 174.6 (78.4–545.1) wide, unlobed, lacking indentations, aseptate (Figure 2A). Haptor almost circular, 1280.2 ± 194.2 (747.2–1613.4) long by 1212.8 ± 234.9 (616.8–1638.2) wide (Figure 2B; Figure 3A). Marginal membrane of haptor 64.3 ± 12.8 (45.2–90.8) wide (Figure 2C). Accessory sclerites 220.8 ± 43.2 (151.6–345.6) long, strong, distally branched (Figure 2B and Figure 3A,B). Anterior hamuli 302.7 ± 77.1 (104.1–435.7) long, relatively stout with sharp, recurved points (Figure 2B,C; Figure 3A,C). Posterior hamuli 126.1 ± 46.1 (83.9–279.8) long, straight, poorly sclerotized with fine recurved points (Figure 2B,C). Marginal hooklets 15.6 ± 6.0 (10.0–20.4) long arranged radially in haptor (Figure 2C and Figure 3A,D). Two pairs of eyes anterior to pharynx. Pharynx 398.4 ± 157.3 (306.1–580.3) long by 485.8 ± 188.3 (384.8–686.1) wide, with six lobes (Figure 2A,D). Intestinal crura extending posteriorly to end of body proper, terminating blindly. Testes 314.2 ± 110.7 (227.8–517.4) long by 251.4 ± 90.4 (177.8–424.0) wide, spherical, lying at midbody, postovarian, lobulated, fenestrated, penetrated by several dorsoventral muscle bundles (Figure 2E). Vas deferens ascending sinistral to ovary and vitelline reservoir, entering penis sac dorsally at level of ootype. Cirrus complex claviform, situated posteriorly to pharynx, consisting of muscular cirrus, prostatic reservoir and seminal vesicle, entire complex enclosed in cirrus pouch (Figure 2D). Ovary 280.7 ± 84.8 (207.9–351.4) long by 334.7 ± 97.0 (250.0–434.3) wide, medial. Vagina absent. Vitelline reservoir transversely wide, anterior to ovary (Figure 2E). Ootype lying posteriorly to cirrus pouch. Uterus short, germiduct joining penis sac toward common genital aperture, which opens sub-terminally just behind left anterior attachment organ. Vitelline follicles extending from behind anterior attachment organs to posterior end of body proper. Egg 128.5 ± 64.0 (98.5–145.7) wide, tetrahedral, with recurved appendages and a slender filament 259.5 ± 139.8 (168.3–336.6) long (Figure 2F).

### 2.2. Molecular Analysis

**Figure 4:**
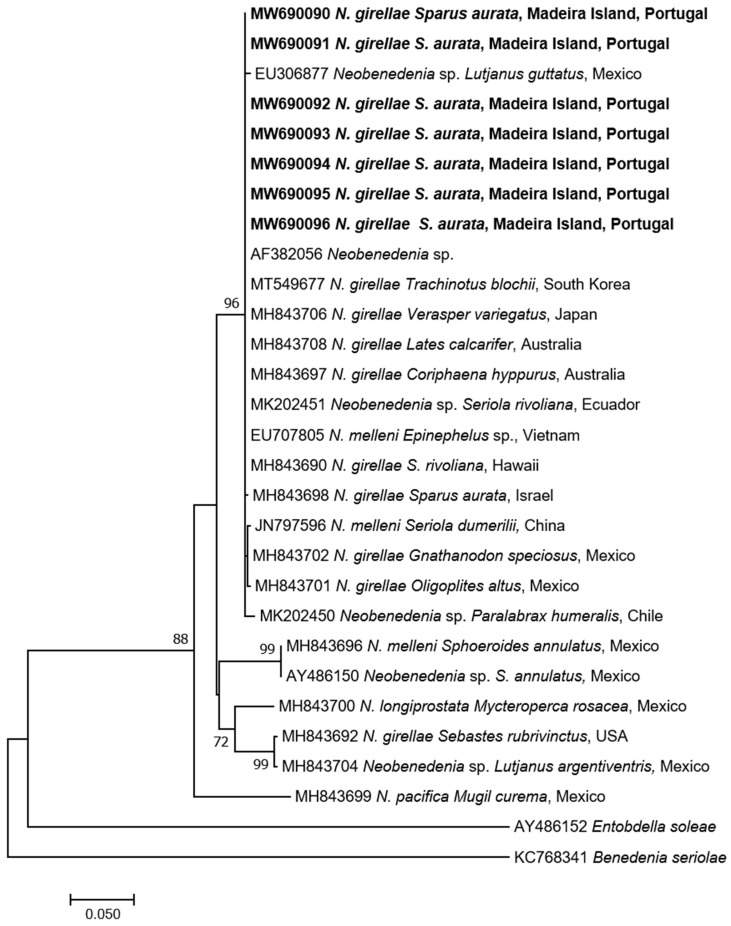
Maximum-Likelihood tree of the 28S rDNA of *Neobenedenia girellae* showing the relationship with the other species of the genus. The tree is drawn to scale, with branch lengths measured in the number of substitutions per site. The newly generated sequences are in bold.

Seven specimens were successfully amplified and sequenced producing 28S amplicons of 1500–1557 bp identical to each other. BLAST search gave the highest identity with *Neobenedenia* sp. (100% AF382056) and *N. girellae* (99.8–99.3%) from several fish species in Australia. The p-distance among *N. girellae* group were 0%, including *Neobenedenia* sp. (EU306877 and MK202450 [21]) from Mexico and Chile respectively, but also *N. melleni* (EU707805 from Vietnam), being most probably misidentified and belonging to *N. girellae* species. The ML tree showed our specimens forming a well-supported cluster with all *N. girellae* from several fish species and different geographical origins (Figure 4).

**Figure 5:**
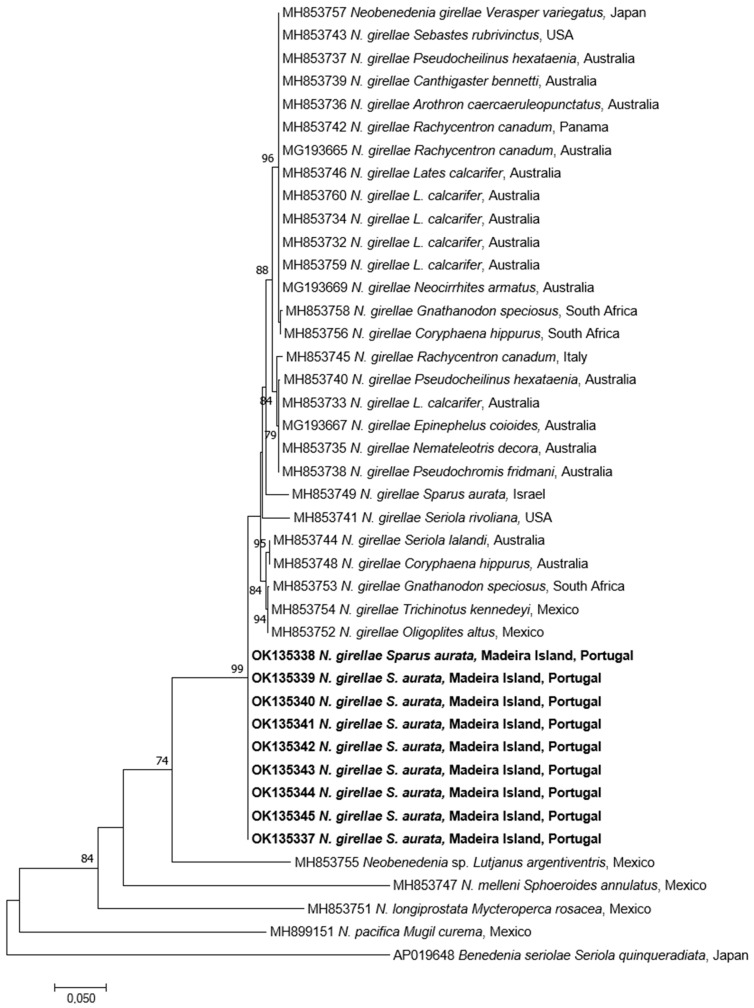
Maximum-Likelihood tree of the *cytB* mtDNA of *Neobenedenia girellae* showing the relationship with the other species of the genus. The tree is drawn to scale, with branch lengths measured in the number of substitutions per site. The newly generated sequences are in bold.

Concerning the *cytB*, nine specimens were successfully amplified and sequenced (704 bp identical to each other). BLAST analysis gave identities ranging from 98.5% to 97.6% with *N. girellae* present in GenBank. The p-distance among the *N. girellae* group were 0.015–0.026. The ML tree showed our specimens included in the *N. girellae* cluster (Figure 5) named Clade A of Brazenor et al. [14] showing the same topology as in the 28S rDNA ML tree.

## 3. Discussion

An increasing amount of evidence coupling morphological and genetic data supports the occurrence of cryptic species within the genus *Neobenedenia* [13]. In the past, *N. girellae* has been synonymized with *N. melleni* based on morphological and host-specificity data [1]. In fact, a morphological revision of the genus *Neobenedenia*, with examination of several specimens of *N. girellae*, failed to identify characters useful to distinguish the latter from *N. melleni,* also due to the fact that specimens of *N. girellae* from different host species show variability in body size, shape and in sclerite morphometry [1]. Since then, the identity of these two species has been debated for several years [22,23] and *N. melleni* has long been considered as the monogenean species with widest host-specificity, being reported from over 100 species of more than 30 families from five orders of captive and wild fish [2,24]. Recently, based on the analysis of nuclear and mitochondrial markers, Brazenor and colleagues [14] demonstrated that *N. girellae* is a separate species to *N. melleni*, as confirmed also by our results, and that a large proportion of samples identified in the past as *N. melleni* may be erroneous; in the light of these findings, *N. girellae* is now considered as the primary monogenean in global fisheries and aquaculture.

In this report we describe the occurrence of *N. girellae* in cultured gilthead seabream, confirming the identity of the parasite by morphological and molecular analyses.

*Neobenedenia girellae* is a widespread pathogen of a variety of cultured teleosts [25] but, with respect to the gilthead seabream, only one report of infection exists [20]. In this report, adult *Sparus aurata* farmed in concrete tanks in Eilat (Red Sea) were experiencing a co-infection with the dinoflagellate *Amyloodinium ocellatum* and *N. melleni*, the first parasite being much more abundant and hyperparasitizing the latter; according to the author, these two parasite species were frequently encountered in *S. aurata* farmed in these conditions in Eilat, particularly during summer. Nevertheless, the species identification of the reported *Neobenedenia* was dubious due to the impossibility of distinguishing *N. melleni* from *N. girellae* based on morphological analysis; more recently, *Neobenedenia* specimens from gilthead seabream from the same area (Eilat) were identified as *N. girellae* with molecular methods [14].

Our work reports for the first time the occurrence of *N. girellae* in cage-reared *S. aurata* from North-eastern Atlantic; in these farming conditions the parasite occurred throughout the year, including fall/winter.

The morphometric data of *N. girellae* analyzed in the present study are in accordance with the previous descriptions of this parasite infecting other fish species [1,26]; however, no morphometric data of specimens from *S. aurata* were available in the literature for comparison. Brazenor et al. [27] highlighted considerable morphological variation, particularly in anterior and posterior attachment organs, between genetically indistinct *N. girellae* infecting different fish species; therefore, data provided in the present work may be useful for future comparative studies. Overall, average measurements of our specimens are slightly smaller than those provided in the original description [26], nevertheless, they fall within the range provided in other morphometric works on *N. girellae* in which the parasite identity has been confirmed by molecular analyses [21,28],

Several fish species from different families are susceptible to *N. girellae* infection: among the species farmed in European waters, the greater amberjack *Seriola dumerili* is reported to be particularly susceptible [17], although no outbreaks have been recorded so far in the Mediterranean Sea and in the Northeastern Atlantic.

Therefore, particular attention should be directed towards the control of imported juveniles from notoriously positive areas (see Whittington [29] and references therein), and to the movement of fish between the Northeastern Atlantic and the adjacent Mediterranean Sea which, to date, has been unaffected; moreover, possible outbreaks could be favoured by high water temperatures of the Mediterranean areas during spring and summer.

Recent evidence highlighted that *N. girellae* undergoes faster life cycle and produces a greater number of eggs with increasing water temperature (30 °C as compared to 20 °C and 25 °C), causing a more severe infection on *S. dumerili*, suggesting the need to apply more frequent measures for controlling the parasites at higher water temperatures [25]. Other susceptible species of commercial interest include the cobia *R. canadum*, the yellowtail *S. quinqueradiata*, the Japanese flounder *P. olivaceus* and spotted halibut *V. variegatus* [10,11,15]. Concerning Sparids, *N. girellae* was reported from the body surface of the red seabream *Pagrus major* cultured in Japan [15], although the infection in this fish host is usually reported as not severe [30]. In susceptible species, *N. girellae* can represent an important agent of mortality [10,12]. During the episodes herein described, the pathological effects of *N. girellae* on the host were not investigated and it was not possible to relate the observed clinical signs, including mortality, to the parasitic infestation due to the concurrent infection with Lymphocystis virus. Nevertheless, our results highlight the possible importance of *N. girellae* as an emerging parasitic infection and a potential threat to *S. aurata* farms and, in general, to European mariculture.

Similarly, to infections due to another pathogenic ectoparasite of cultured gilthead seabream, the Microcotylid *Sparicotyle chrysophrii* (Van Beneden & Hesse, 1863) Mamaev, 1984, culture nets represent a good substrate for the adhesion of *Neobenedenia* eggs which have a long filamentous appendage [10,31]; this feature could favour the adaptation of the parasite to the sea cage farming system widely adopted throughout the Mediterranean Sea for the culture of *S. aurata*.

Concerning the possible origin of the parasite in the investigated farm, our findings suggest a possible risk of parasite transfer between different fish species: in one of the two sites sampled for the present study, severe infections with capsalids had been reported in cultured *S. dumerili* two years before, although the identity of parasites involved in the previous outbreak had not been confirmed. Furthermore, wild *S. dumerili* have been observed in the proximity of the floating cages under investigation, however parasitological data from wild *Seriola* and other fish species are currently not available.

As our findings suggest, it is of critical importance to strengthen controls on imported batches of susceptible fish species and to study the evolution of this parasitic infection in order to assess the possible threat to Mediterranean aquaculture and to monitor the presence of *N. girellae* in different species of wild fish occurring around the floating cages in order to assess the risk of parasite transfer to farmed *S. aurata* and vice-versa.

## 4. Materials and Methods

Recurrent episodes of skin parasitic infections due to Capsalid monogeneans were recorded in gilthead seabream during routine veterinary checks throughout the year 2019 from two different sites of a fish farm off Madeira Island (NE Atlantic) approximately 40 km apart from each other. The parasitological findings are not from a targeted epidemiological survey but represent observations carried out during field diagnostic activities. Therefore, the quantitative parasitological data here reported refer to the whole sampling carried out in the two farm facilities (A and B); in particular in February (water temperature 18 °C), August (24–25 °C), October (23 °C) and December (20 °C) 204 gilthead seabream from facility A (from 8 to 304 g; 135 fish ≤30 g and 69 fish >30 g) and in August 24 gilthead seabream from facility B (around 100 g).

Fifteen parasites were collected, fixed in 70% ethanol and then forwarded to the fish pathology lab (DIMEVET) for identification. All the specimens were subjected to microscopic observation following clarification in Amman’s lactophenol in order to study their taxonomic features. Before clearing the parasites, a section of the body without diagnostic characters was excised with a sterile scalpel and stored at −20 °C for further molecular analysis.

Measurements were taken with the imaging software NIS-Elements (Nikon, Campi Bisenzio (FI), Italy), and are given in micrometers (µm; mean ± Standard Deviation followed by the range in parentheses). All the specimens are stored in our private parasitological collection and are available for examination.

For Scanning Electron Microscopy, fixed specimens were dehydrated in a graded ethanol series, critical point dried and sputter coated with gold-palladium, and observed using a Phenom XL G2 Desktop SEM (Thermo Fisher Scientific, Eindhoven, The Netherlands) operating at 5 kV.

For molecular analysis, genomic DNA was extracted from nine worms using PureLink^®^ Genomic DNA Kit (Life Technologies, Carlsbad, CA, USA) following the manufacturer’s instructions. The amplification of the 28S rDNA region was performed with primers U178_f (5′-GCACCCGCTGAAYTTAAG-3′) and L1642_r (5′-CCAGCGCCATCCATTTTCA-3′) [32]. The thermal cycler program (Tpersonal, Biometra, Göttingen, Germany) was 40 cycles of 30 s at 94 °C, 30 s at 52 °C and 2 min at 72 °C, preceded by a denaturation step at 94 °C for 2 min and followed by an extended elongation step at 72 °C for 10 min. The PCR products were electrophoresed on 1% agarose gel stained with SYBR^®^ Safe DNA Gel Stain (Invitrogen, Thermo Fisher Scientific, Carlsbad, CA, USA) in 0.5X TBE. Amplicons were purified by Nucleo-Spin Gel and PCR Cleanup (Mackerey-Nagel, Düren, Germany). Samples were sequenced with the internal primers 900F (5′-CCGTCTTGAAACACGGACCAAG-3′) and EDC2 (5′-CCTTGGTCCGTGTTTCAAGACGGG-3′) [32]. The *cytB* mtDNA was amplified using the primers and protocol reported by Brazenor et al. [14].

All samples were sequenced with an ABI 3730 DNA analyzer (StarSEQ, Mainz, Germany). The DNA trace files were assembled with ContigExpress (VectorNTI Advance 11 software, Invitrogen, Carlsbad, CA, USA) and the consensus sequences were compared with previously published data by using BLAST tools (https://blast.ncbi.nlm.nih.gov/Blast.cgi assessed on 9 September 2021). Our sequences were multiple aligned with the ones retrieved from GenBank, using BioEdit 7.2.5 [33]. Pairwise distance and maximum likelihood (ML) tree (HKY+G for 28S rDNA and GTR+G+I for Cytb, bootstrap of 1000 replicates for both) were obtained by MEGA version X [34]. The sequences generated in this study were deposited in GenBank under the accession numbers MW690090-96 (28S rDNA) and OK135337-45 (*cytB* mtDNA).

## Data Availability

Not applicable.
